# Variable organization of symbiont-containing tissue across planthoppers hosting different heritable endosymbionts

**DOI:** 10.3389/fphys.2023.1135346

**Published:** 2023-03-22

**Authors:** Anna Michalik, Diego Castillo Franco, Junchen Deng, Teresa Szklarzewicz, Adam Stroiński, Michał Kobiałka, Piotr Łukasik

**Affiliations:** ^1^ Department of Developmental Biology and Morphology of Invertebrates, Institute of Zoology and Biomedical Research, Faculty of Biology, Jagiellonian University, Krakow, Poland; ^2^ Institute of Environmental Sciences, Faculty of Biology, Jagiellonian University, Krakow, Poland; ^3^ Doctoral School of Exact and Natural Sciences, Jagiellonian University, Kraków, Poland; ^4^ Museum and Institute of Zoology, Polish Academy of Sciences, Warsaw, Poland

**Keywords:** planthoppers, nutritional endosymbiosis, bacteriome, symbionts, symbiont replacement

## Abstract

Sap-feeding hemipteran insects live in associations with diverse heritable symbiotic microorganisms (bacteria and fungi) that provide essential nutrients deficient in their hosts’ diets. These symbionts typically reside in highly specialized organs called bacteriomes (with bacterial symbionts) or mycetomes (with fungal symbionts). The organization of these organs varies between insect clades that are ancestrally associated with different microbes. As these symbioses evolve and additional microorganisms complement or replace the ancient associates, the organization of the symbiont-containing tissue becomes even more variable. Planthoppers (Hemiptera: Fulgoromorpha) are ancestrally associated with bacterial symbionts *Sulcia* and *Vidania*, but in many of the planthopper lineages, these symbionts are now accompanied or have been replaced by other heritable bacteria (e.g., *Sodalis*, *Arsenophonus*, *Purcelliella*) or fungi. We know the identity of many of these microbes, but the symbiont distribution within the host tissues and the bacteriome organization have not been systematically studied using modern microscopy techniques. Here, we combine light, fluorescence, and transmission electron microscopy with phylogenomic data to compare symbiont tissue distributions and the bacteriome organization across planthoppers representing 15 families. We identify and describe seven primary types of symbiont localization and seven types of the organization of the bacteriome. We show that *Sulcia* and *Vidania*, when present, usually occupy distinct bacteriomes distributed within the body cavity. The more recently acquired gammaproteobacterial and fungal symbionts generally occupy separate groups of cells organized into distinct bacteriomes or mycetomes, distinct from those with *Sulcia* and *Vidania*. They can also be localized in the cytoplasm of fat body cells. Alphaproteobacterial symbionts colonize a wider range of host body habitats: *Asaia*-like symbionts often colonize the host gut lumen, whereas *Wolbachia* and *Rickettsia* are usually scattered across insect tissues and cell types, including cells containing other symbionts, bacteriome sheath, fat body cells, gut epithelium, as well as hemolymph. However, there are exceptions, including *Gammaproteobacteria* that share bacteriome with *Vidania*, or *Alphaproteobacteria* that colonize *Sulcia* cells. We discuss how planthopper symbiont localization correlates with their acquisition and replacement patterns and the symbionts’ likely functions. We also discuss the evolutionary consequences, constraints, and significance of these findings.

## 1 Introduction

Intimate symbiotic relationships with microorganisms have played significant roles in the biology of many insects, influencing their nutrition, reproduction, development, protection against antagonists, and susceptibility to toxin resistance (Baumann, 2005; Flórez et al., 2015). Symbiosis with microbes is also among the crucial drivers of insects’ evolutionary diversification and adaptation to different environmental and biotic challenges associated with diverse global ecosystems (Feldhaar, 2011). Insect symbioses may involve both prokaryotic and eukaryotic microorganisms and differ in complexity, stability, and degree of interdependence between host and symbionts (Buchner, 1965; Douglas, 2016; McCutcheon et al., 2019; Frago et al., 2020). Probably, the most common way of describing the diversity of these symbioses is by dividing them into those that live outside of the body cavity - within the gut or on the cuticle (ectosymbionts), and those within the body cavity, in tissues and cells (endosymbionts). Endosymbioses are further partitioned into facultative and obligate. Facultative endosymbionts are not essential to hosts but can alter their biology in multiple ways, including by manipulating reproduction or providing ecological benefits, including protection against natural enemies or extreme temperatures. Their net fitness effects can range from mutualism to parasitism, depending on the conditions. Obligate endosymbionts are strictly necessary for the host insects, as they provide essential amino acids and vitamins deficient in their diet, and these nutritional provisions are indispensable for host growth and reproduction.

These different types of symbioses vary in the stability of their association with the host. Ectosymbioses vary dramatically in that respect, with some acquired from the environment each generation and others having evolved a variety of mechanisms of maternal or social transmission (Salem et al., 2015). Facultative endosymbioses are transmitted through the female reproductive system, with high but sometimes not perfect fidelity but are also capable of occasional transmission across host lines and species. The obligate endosymbionts are transmitted across host generations strictly maternally (Douglas, 2016; Perreau and Moran, 2021). Some of them, including many fungi or *Gammaproteobacteria*, have been acquired relatively recently (Husnik and McCutcheon, 2016; Matsuura et al., 2018; Michalik et al., 2021), while others can maintain the relationship for hundreds of millions of years (Moran et al., 1993; Bennett and Moran, 2013). In some cases, co-obligate symbioses exist when two or more symbionts, independently acquired at different times, complement the nutritional function of each other (Douglas, 2016).

The age of association and the level of symbiont integration into host biology is linked to the nature and organization of host organs where these symbionts reside. Facultative or newly-acquired symbionts often disperse in diverse tissues. When the relationship becomes tighter, the localization and organization of endosymbionts in the host body also change. Ancient, obligate nutrient-providing endosymbionts often reside in most sophisticated, dedicated organs made up of specialized insect cells. Depending on whether the symbiont is a bacterium or a fungus, these organs are termed bacteriomes or mycetomes, respectively, and the cells they are built from are referred to as bacteriocytes or mycetocytes (Buchner, 1965); in this text, for simplicity, we will write about bacteriomes/bacteriocytes, but most information applies to fungus-hosting organs as well. The main role of bacteriocytes is to mediate in exchanging the metabolites between symbionts and hosts and control the symbiont population size (Smith and Moran, 2020). As bacteriocytes repeatedly evolved in many insect lineages, bacteriome organization varies between host clades and often also among different symbionts that live in the same host (Buchner, 1965). The newly-acquired microorganisms can live both within bacteriomes and other tissues. The compartmentalization of symbionts into host cells allows them, on the one hand, to shelter from the insect’s immune system cells; on the other hand, it ensures the host control of the symbiont population growth (Harris et al., 2010). In multi-symbiotic systems, the spatial arrangement of obligate, nutritional symbionts may also reflect the metabolic convergence between them. Co-obligate symbionts that exchange the metabolites in the biosynthesis processes (e.g., synthesis of amino acids or vitamins) are more likely to be gathered within the same bacteriome, whereas symbionts not sharing biosynthesis pathways are often localized in distinct organs (Douglas, 2016). Additionally, as symbioses evolve and ancient symbiotic associates get complemented or replaced by others, the organization of the symbiont-containing tissue is likely to change further. However, little is known about the localization of newly-acquired microorganisms or how it is determined, even though it significantly influences the outcome of the symbiotic interaction.

One of the best insect groups to study symbiont localization is Auchenorrhyncha - a suborder of hemipterans with particularly complex symbiotic systems. The ancestral symbiont of all Auchenorrhyncha is *Sulcia muelleri* (Bacteroidetes; hereafter *Sulcia*), which colonized the common ancestor of these insects ca. 300 mya and has co-diversified with the hosts since then (Moran et al., 2005). *Sulcia* is always accompanied by additional microbes, for example, with alphaproteobacterium *Hodgkinia* in cicadas (McCutcheon et al., 2009; McCutcheon and Moran, 2010) or betaproteobacteria *Zinderia* in spittlebugs and *Vidania* in planthoppers (McCutcheon and Moran, 2010; Urban and Cryan, 2012). However, these symbioses are far from stable, with the relatively frequent acquisition of new microbes and symbiont replacement driving additional changes (Sudakaran et al., 2017). Well-known examples of the replacement of an ancient symbiont include *Sodalis* instead of *Zinderia* in Philaenini spittlebugs (Koga et al., 2013) and repeated replacements of *Hodgkinia* by *Ophiocordyceps* fungi in various lineages of cicadas (Matsuura et al., 2018; Huang et al., 2020; Huang et al., 2023). Additional co-infecting microbes, especially *Gammaproteobacteria* related to *Sodalis* and *Arsenophonus*, have also been reported from many Auchenorrhyncha lineages and are typically linked to nutrition (Michalik et al., 2021)—although, in most cases, evidence is lacking, and the nature and stability of these associations unclear (Michalik et al., 2014a; Kobiałka et al., 2016).

Planthoppers (Fulgoromorpha) are an ecologically and evolutionarily diverse Auchenorrhyncha group encompassing almost 14,000 known species representing 21 extant families (Bourgoin, 2022). They represent various degrees of trophic specificity and ecological relationships. Due to their food preferences and modes of feeding, planthoppers are vectors of plant pathogens, and several species are considered serious agricultural pests (Wilson and O’Brien, 1987). We know that these insects have been ancestrally associated with bacterial symbionts *Sulcia* and *Vidania* (Urban and Cryan, 2012; Bennett and Mao, 2018; Michalik et al., 2018a; Michalik et al., 2021). However, in many planthopper lineages, they are now accompanied or have been replaced by other heritable bacteria (e.g., *Sodalis*, *Arsenophonus*, *Purcelliella*, *Wolbachia*) or fungi (Michalik et al., 2009; Bennett and Mao, 2018; Michalik et al., 2021). These associations and modes of their transmission can be very diverse, prompting Paul Buchner, after decades of observations, to famously describe them as “a veritable fairyland of symbiosis” (Buchner, 1965). However, our understanding of the diversity and evolution of these associations is grossly incomplete. Before the Second World War, Hans Müller and Paul Buchner have characterized many planthopper-symbiont associations using histological techniques and interpreted the patterns with impressive accuracy, but lacked tools to verify their identity (Müller, 1940a; Müller, 1940b; Buchner, 1965). More recently, sequencing-based approaches have provided information about the identity of some of these microbes (Bennett and Mao, 2018; Michalik et al., 2021), but we know much less about their genomics characteristics, evolutionary patterns, or functions. Importantly, microscopy and sequencing surveys have not generally been combined, and the use of modern microscopy techniques is limited to a few taxa (Michalik et al., 2021). Hence, we lack the link between the host and symbiont identity, the organization of symbiont-holding tissue, and symbiosis functions.

Here, we aimed to fill this knowledge gap by exploring the symbiotic systems of 44 planthoppers species of 15 families, representing the main evolutionary lineages of modern Fulgoromorpha. We combined light, fluorescence, and transmission electron microscopy with phylogenomic data to compare the bacteriomes’ organization across planthoppers hosting different symbiont combinations. This approach allowed us to identify and describe seven different categories of symbiont localization within the host insect’s body and seven types of bacteriome organization, which reflects the diversity of anatomical integrations of endosymbiotic associations that have evolved in insects. We emphasized the localization of microorganisms complementing the ancestral symbionts and newly-acquired microorganisms that replaced the ancestral associates.

## 2 Materials and methods

### 2.1 Insect collection and preservation

Taxonomic sampling included 44 planthopper species representing 15 families, including Acanalonidae (one species), Achilidae (two species), Caliscelidae (two), Cixiidae (three), Delphacidae (seven), Derbidae (three), Dictyopharidae (seven), Fulgoridae (three), Flatidae (two), Issidae (six), Lophopidae (one), Meenoplidae (one), Ricanidae (three), Tettigometridae (two), and Tropiduchidae (one). Adult insects were collected in Bulgaria, Italy, Vietnam, and Poland between 2014 and 2019. After sampling, the material was preserved in an appropriate fixative (96%–100% ethanol or 2.5% glutaraldehyde) and stored at 4°C until further processing. Sampling details are summarized in Supplementary Table S1. Representative specimens from each species were identified based on morphological characteristics.

### 2.2 Sequencing-based symbiont survey

#### 2.2.1 DNA extraction and metagenomic library preparation and sequencing

DNA was extracted from dissected bacteriomes or insect abdomens using one of three different DNA extraction kits: Sherlock AX isolation kit (A&A Biotechnology, Poland), Bio-Trace DNA Purification Kit (Eurx, Poland), and Genomic Mini AX Yeast Spin kit (A&A Biotechnology, Poland), according to manufacturers’ protocols. The metagenomic libraries for high-throughput sequencing on the Illumina platform were prepared using NEBNext Ultra II FS DNA Library Prep and NEBNext DNA Ultra II kits, with a target insert length of 350 bp. The pooled libraries were sequenced on Illumina HiSeq X or NovaSeq 6000 S4 lanes (2 × 150 bp reads).

#### 2.2.2 Metagenome-based reconstruction of microbiome composition

The taxonomic composition of the microbial symbiont community was assessed based on the sequences of small subunit rRNA genes (16S rRNA and 18S rRNA). We reconstructed rRNA sequences using PhyloFlash v3.4 (Gruber-Vodicka et al., 2020), using the option -- everything, including EMIRGE approach (Miller et al., 2010). The taxonomic classification was performed against a customized SILVA database (v138), which included several so-far-unpublished sequences of planthopper symbionts. The results were manually verified through comparisons of sequences and symbiont identities among related species and microscopy and marker gene-sequencing datasets for the additional specimens (data not shown).

#### 2.2.3 Host mitogenome assembly and phylogenetic analysis

The metagenomic reads were assembled using MEGAHIT v1.1.3 (Li et al., 2016) with k-mer size from 99 to 255. The host mitochondrial contigs were identified using “blastn” and “blastx” searches against custom databases, which included DNA and amino acid sequences of published planthopper mitogenomes. The identified mitochondrial contigs were then annotated with a custom Python script modified from Łukasik et al. (2019). The script first extracted all the Open Reading Frames (ORFs) and their amino acid sequences from a genome. Then, the ORFs were searched recursively using HMMER v3.3.1 (Eddy, 2011), through custom databases containing manually curated sets of protein-coding and rRNA genes of planthopper mitochondria. rRNA genes were searched with nhmmer (HMMER V3.3.1) (Wheeler and Eddy, 2013), and tRNAs were identified with tRNAscan-SE v2.0.7 (Chan et al., 2021). For phylogenetic reconstructions, we used concatenated alignments of 13 mitochondrial protein-coding genes (*nad2*, *cox1*, *cox2*, *atp8*, *atp6*, *cox3*, *nad3*, *nad5*, *nad4*, *nad4L*, *nad6*, *cob*, and *nad1*) and one mitochondrial ribosomal RNA (*rrnL*), resulting in a total dataset length of 11,713 bp.

The maximum likelihood tree of host species was constructed in IQ-Tree on XSEDE (Minh et al., 2020) and implemented in CIPRES v.3.3 (Miller et al., 2010). “Model Selection” (Kalyaanamoorthy et al., 2017) was selected to search for the best model in CIPRES. The partition type was set to allow the 14 partitions (one for each marker) to allow different evolutionary rates (Chernomor et al., 2016). “TESTNEWMERGE” was specified to allow partitions with similar speed to be analyzed as a single partition. The best fit models were decided by the highest BIC (Bayesian Information Criterion) scores. Bootstrapping was conducted using “SH-aLRT” bootstrap methods with 1,000 replicates. All other options were set to default.

#### 2.2.4 Amplicon-based reconstruction of intraspecific microbiome diversity

For selected species that were represented by multiple individuals in our collection, we sequenced amplicons for the V4 region of the 16S rRNA gene as a means of assessing intra-species microbiome diversity, alongside the host mitochondrial cytochrome oxidase I (COI) gene as a means of confirming host identity. We described the laboratory workflows previously (Michalik et al., 2021). Briefly, we used a two-step PCR library preparation protocol, where in the first round of PCR, we simultaneously amplified marker regions of interest using template-specific primers 515F/806R and COIBF3/COIBR2 with Illumina adapter stubs, and then used bead-purified PCR products as the template for the second, indexing PCR. Pooled libraries were sequenced on an Illumina MiSeq v3 lane (2 × 300-bp reads) at the Institute of Environmental Sciences of Jagiellonian University. We processed amplicon data separately for both targeted regions using custom pipeline based on USEARCH/VSEARCH (Edgar, 2010; Rognes et al., 2016). Reads assembled into contigs were quality-filtered, then dereplicated and denoised, aligned against the reference databases, screened for chimeras using UCHIME (Edgar et al., 2011), classified taxonomically, and finally, clustered at 97% identity level using the nearest-neighbor algorithm and divided into OTUs. The COI data were used to confirm insect species identity, while data on relative abundance of symbiont types was visualized using R v. 4.0.2 (R Development Core Team) with the ggplot2 package (Wickham, 2011).

### 2.3 Microscopic analyses

#### 2.3.1 Histological and ultrastructural analyses

Adult specimens of each species were dissected partially in the field and immediately preserved in 2.5% glutaraldehyde in 0.1 M phosphate buffer (pH 7.2). In the laboratory, the fixed material was rinsed three times in the same phosphate buffer with the addition of sucrose (5.8 g/100 mL) and then postfixed in 1% osmium tetroxide for 2 h at room temperature. Next, samples were dehydrated in the graded series of ethanol (30%–100%—three times 15 min in each concentration) and acetone (three times for 10 min) and embedded in epoxy resin Epon 812 (Merck, Darmstadt, Germany). The resin blocks were cut using Reichert-Jung ultracut E microtome into semithin (1 um thick) and ultrathin (90 nm thick) sections. Semithin sections were stained with 1% methylene blue in 1% borax and examined using the Nikon Eclipse 80i light microscope. Ultrathin sections were contrasted with uranyl acetate and lead citrate and analyzed using JEOL JEM 2100 electron transmission microscope.

#### 2.3.2 Fluorescence *in situ* hybridization

The fluorescence *in situ* hybridization was performed with symbiont-specific probes complementary to their 16S rRNA gene sequences (see Supplementary Table S2). Insects preserved in ethanol were rehydrated and then postfixed in 4% paraformaldehyde for 2 hours at room temperature. Next, the material was dehydrated again by incubation in increased concentrations of ethanol (30%–100%) and acetone (30 min for each concentration), embedded in Technovit 8,100 resin (Kulzer, Wehrheim, Germany), and cut into semithin sections (1 um thick). The sections were then incubated overnight at room temperature in a hybridization buffer containing the specific sets of probes with the final concentration of 100 nM (see Supplementary Table S2). After hybridization, the slides were washed in PBS three times, dried, covered with ProLong Gold Antifade Reagent (Life Technologies), and examined using a confocal laser scanning microscope Zeiss Axio Observer LSM 710.

## 3 Results

### 3.1 High-throughput sequencing reveals the diversity of planthopper symbioses

**FIGURE 1:**
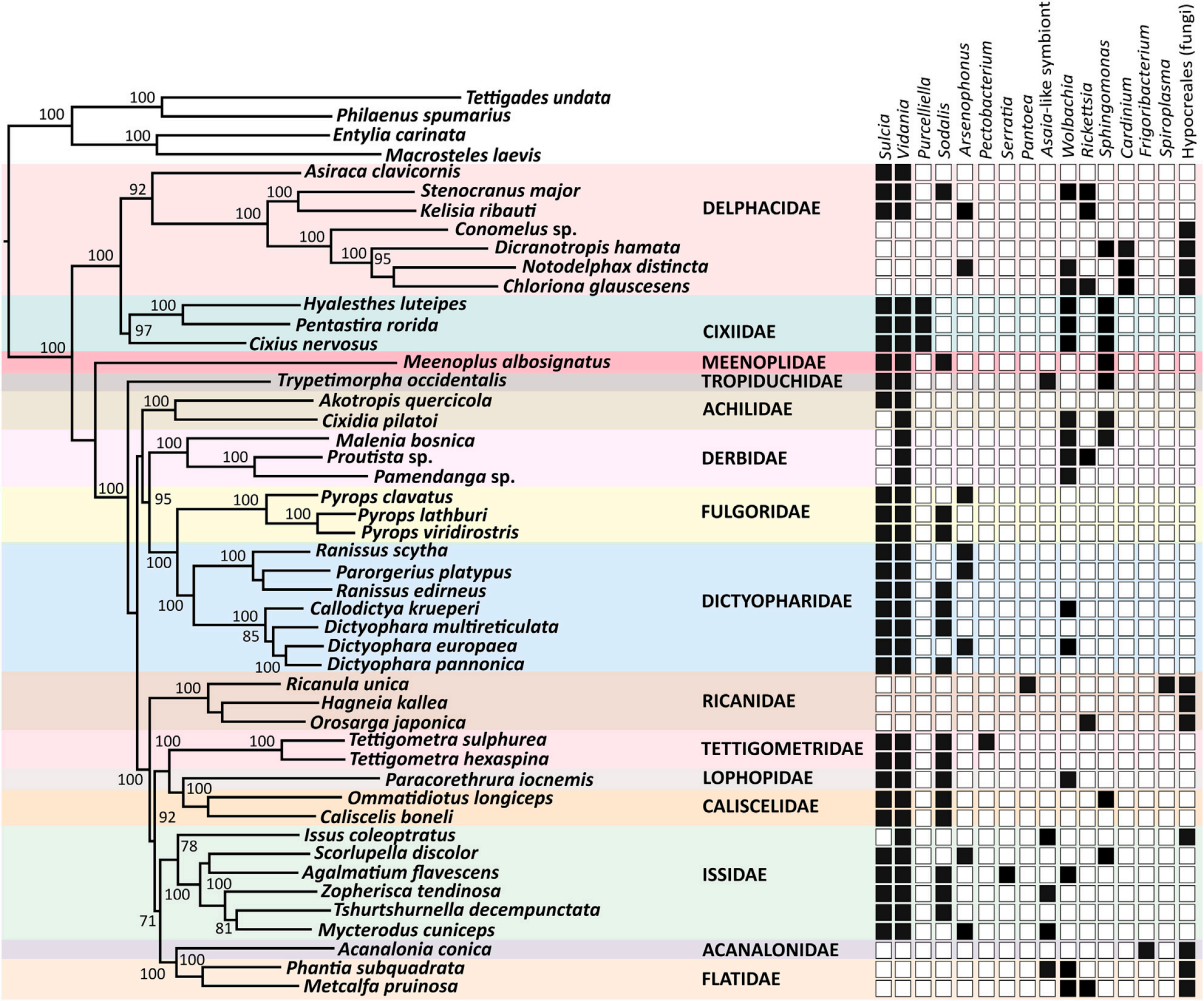
Symbiont diversity across planthopper phylogeny. The Maximum Likelihood phylogeny is constructed based on concatenated 13 mitochondrial protein-coding genes and one mitochondrial ribosomal RNA, of a total size of 11,713 bp. Bootstrap support values > 70% are shown. The heatmap shows the presence/absence of planthopper symbiont genera based on the sequences of 16S rDNA genes reconstructed from metagenomic datasets.

The reconstruction of microbial community composition based on metagenome-derived, full-length bacterial 16S rRNA and fungal 18S rRNA sequences revealed a striking variety of symbioses across 44 sampled species from 15 planthopper families (Figure 1). Specifically, Phyloflash recovered 16S rRNA sequences of 15 bacterial genera representing four phyla: *Bacteroidetes* (*Sulcia*, *Cardinium*), *Proteobacteria* (*Vidania*, *Purcelliella*, *Sodalis*, *Arsenophonus*, *Wolbachia*, *Rickettsia*, *Pectobacterium*, *Asaia*-like symbionts, *Serratia*, *Sphingomonas*, and *Pantoea*), *Actinobacteria* (*Frigoribacterium*), and *Tenericutes* (*Spiroplasma*). It also reconstructed sequences of fungi in the order *Hypocreales* (phylum *Ascomycota*). Generally, symbiont community composition in Fulgoromorpha is linked to host phylogeny, but with some exceptions, including families Achilidae and Issidae.

The most common bacterial symbiont in Fulgoromorpha is the ancient betaproteobacterial nutritional endosymbiont *Vidania*, detected in 34 species representing 12 families. In 9 of these families, including Caliscelidae, Cixiidae, Delphacidae, Dictyopharidae, Fulgoridae, Lophopidae, Meenoplidae, Tettigometridae, and Tropiduchidae, *Vidania* always co-resides with *Sulcia*. In contrast, in the families Achilidae and Issidae, some species host both these ancestral symbionts while others only harbor *Vidania*. Derbidae is the only family where all tested representatives lack *Sulcia* but harbor *Vidania*. In all examined species of families Acanalonidae, Flatidae, Ricaniidae, and some members of the family Delphacidae (subfamily Delphacinae), we did not observe *Sulcia* or *Vidania*, and detected fungal symbionts belonging to the order *Hypocreales* instead (Figure 1).

Planthoppers hosting ancestral symbionts *Sulcia* and *Vidania* are associated with at least one additional bacterium, which in most species belong to the class *Gammaproteobacteria*. Among them, *Sodalis* and *Arsenophonus* are the most common, colonizing 16 and 8 species in 8 and 4 families, respectively. Another gamma-symbiont, *Purcelliella*, occurs exclusively in members of the family Cixiidae. Other gammaproteobacterial associates found in some examined planthoppers include *Pectobacterium*, *Serratia,* and *Pantoea*. Fulgoromorpha are also frequently colonized by microbes belonging to the class *Alphaproteobacteria*. *Wolbachia* and/or *Rickettsia,* known as facultative endosymbionts of diverse insects, are detected in 8 out of 15 families. In 5 species: *Issus coleoptratus*, *Mycterodus cuniceps*, *Phantia subquadrata*, *Trypetimorpha occidentalis*, and *Zopherisca tendinosa*, we also identified alphaproteobacteria from the family Acetobacteraceae. Bacteria limited to species harboring fungal symbionts represent the genera *Cardinium* (in *Dicranotropis hamata* and *Notodelphax distincta*), *Frigoribacterium* (in *Acanalonia conica*), and *Spiroplasma* (in *Ricanula unica*) (Figure 1).

**FIGURE 2:**
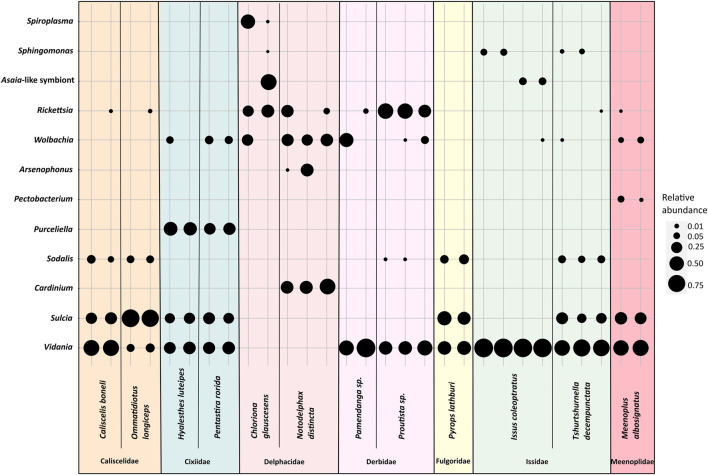
The diversity of bacterial symbionts in replicate individuals of selected planthopper species. The blob sizes correspond to the relative abundance of a symbiont from a given bacterial genus.

In some of 30 planthopper species where multiple individuals per population were used for amplicon sequencing, we also observed intra-species diversity in the microbiome composition (Figure 2). *Sulcia*, *Vidania,* and fungal symbionts were uniformly either present or absent in all individuals of a species. In contrast, in some species, we observed differences in the composition of additional symbionts (e.g., *Wolbachia*, *Rickettsia,* and *Sphingomonas*) and their relative abundance between individuals sampled from one population. For example, *Asaia*-like symbiont was detected in only 2 out of 3 individuals of *I. coleoptratus,* while the third hosted *Sphingomonas* but lacked *Asaia.*

### 3.2 Microorganisms associated with planthoppers show different localization patterns

**FIGURE 3:**
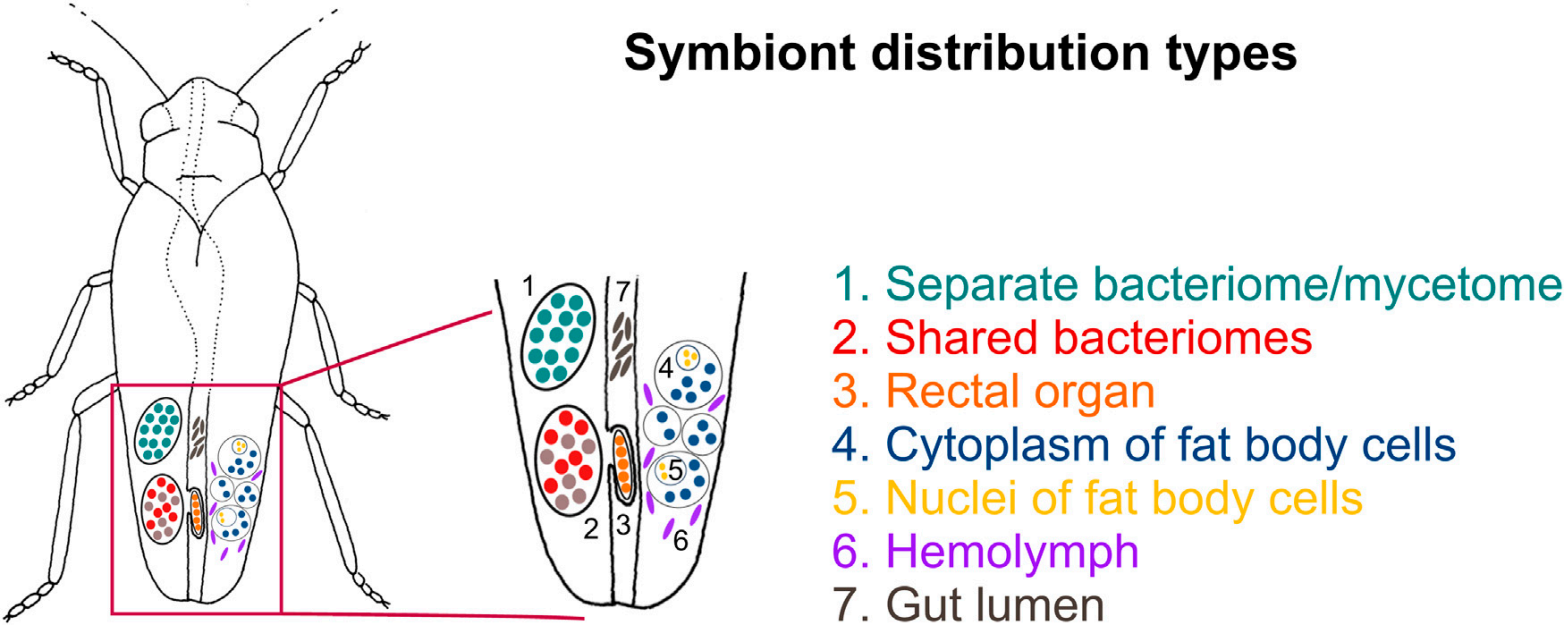
Schematic representation showing the possible symbiont localizations in the host-insect body. For a detailed description of symbiont localizations, see the main text.

**FIGURE 4:**
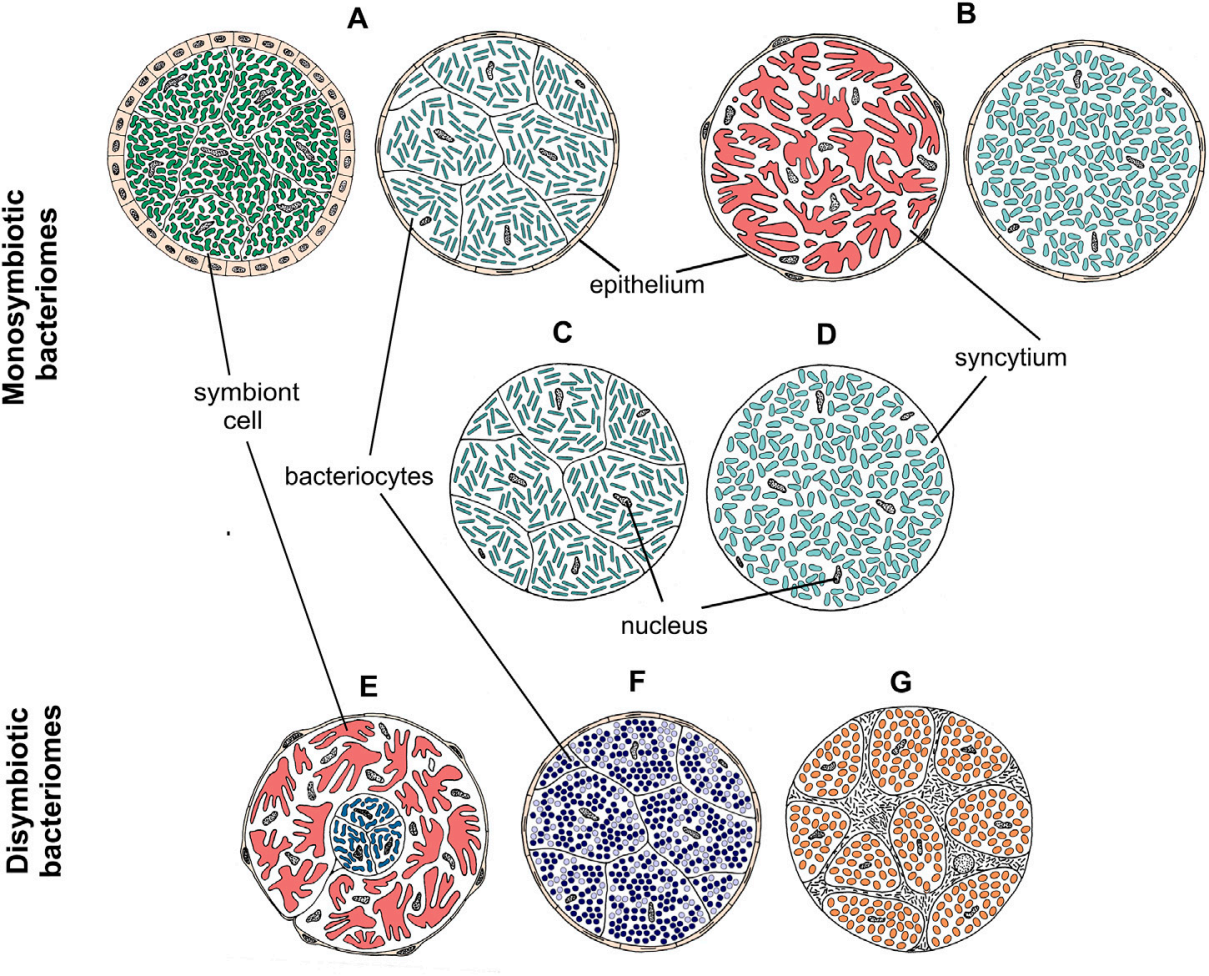
Drawings showing the organization of different types of bacteriomes and mycetomes in planthoppers. **(A–D)** Monosymbiotic bacteriomes, **(E–G)** Disymbiotic bacteriomes. For a detailed description of bacteriome categories, see the main text.

Our broad microscopic investigations have revealed that symbionts associated with planthoppers are distributed in very different ways across host insect tissues (Figure 3). Most planthopper symbionts reside in specialized organs (bacteriomes or mycetomes), which can differ considerably in their organization depending on microorganisms hosted (Figure 4). Below, we present different symbiont tissue localizations and then describe different bacteriome/mycetome types. In subsequent sections, we explain how different symbiont taxa are distributed across these localizations and symbiont-containing organ types in different planthopper clades.

We identified seven distinct localizations of symbionts (Figure 3; Table 1). Most often, (1) different symbionts are segregated to separate bacteriomes; however, in some cases, (2) they share a common bacteriome. In females only, in addition to the bacteriomes localized in the body cavity, (3) a single bacteriome called rectal organ occurs in the deep invagination of the hindgut. Planthopper symbionts may also be distributed in the fat tissue, where they may reside in (4) the cytoplasms of the fat body cells or (5) fat body cell nuclei. Besides intracellular localization, microorganisms associated with planthoppers may occur extracellularly: either (6) in the hemolymph between fat body cells or (7) in the gut lumen (Table 1).

**TABLE 1:** Symbiont tissue localizations and bacteriome types identified among the surveyed planthoppers.

Distribution type	Symbiont	Taxonomy	Bacteriome type	Host species/family where observed
1. Separate bacteriome/mycetome	*Sulcia*	Bacteroidetes: Flavobacteriales	A	Always when *Sulcia* present, except when joined by another symbiont (see 2)
	*Vidania*	Betaproteobacteria	B	Always when *Vidania* present, except when joined by another symbiont (see 2)
	*Sodalis*	Gammaproteobacteria: Enterobacterales	A	Always when present, except for co-infections (see 2)
	*Arsenophonus*	Gammaproteobacteria: Enterobacterales	C	Always when present, except for co-infections (see 2)
	Fungal symbionts	Fungi: Ascomycota: Hypocreales	B, D	Flatidae
2. Shared bacteriomes	*Purcelliella* (accompanies *Vidania*)	Gammaproteobacteria: Enterobacterales	E	exclusively Cixiidae
	*Sodalis* (with *Pectobacterium*)	Gammaproteobacteria: Enterobacterales	G	*Tettigometra sulphurea*
	*Pectobacterium* (with *Sodalis*)	Gammaproteobacteria: Enterobacterales	G	*Tettigometra sulphurea*
	*Arsenophonus* (with *Vidania* or *Sulcia*)	Gammaproteobacteria: Enterobacterales	E, F	*Pyrops clavatus* (type E); S*corupella discolor* (type F)
	*Acetobacteraceae* (*Asaia*-like) (accompanies *Sulcia*)	Alphaproteobacteria: Rhodospirillales	G	*Trypetimorpha occidentalis*
	*Wolbachia* (with *Vidania* or *Sulcia*)	Alphaproteobacteria: Rickettsiales	F	*Callodictya krueperi*, *Dictyophara europaea*, Derbidae, Cixiidae
3. Rectal organ	*Vidania*	Betaproteobacteria	A	Always when *Vidania* is present - in females only
4. Cytoplasm of fat body cells	*Wolbachia*	Alphaproteobacteria: Rhodospirillales	-	*Stenocranus major*, *Notodelphax distincta*, *Cixidia pilatoi*, *Paracorethrura iocnemis*, *Agalmatium flavescens*, *Phantia subquadrata*, *Metcalfa pruinosa*, Derbidae
	*Rickettsia*	Alphaproteobacteria: Rickettsiales	-	Always when *Rickettsia* present
	Fungal symbionts	Fungi: Ascomycota: Hypocreales	-	*Notodelphax distincta*, *Issus coleoptratu*s, Ricaniidae
5. Nuclei of fat body cells	*Rickettsia*	Alphaproteobacteria: Rickettsiales	-	*Orosanga japonica*
6. Hemolymph	Fungal symbionts	Fungi: Ascomycota: Hypocreales	-	*Orosanga japonica*
7. Gut lumen	*Acetobacteraceae* (*Asaia-*like)	Alphaproteobacteria: Rhodospirillales	-	*Issus coleoptratus*, *Zopherisca tendinosa*, *Mycterodus cuniceps*, *Phantia subquadrata*

The most common localization of planthopper symbionts are bacteriomes—structures usually consisting of bacteriocytes filled with symbionts. Analogous organs filled with fungal cells are known as mycetomes/mycetocytes, but as their general organization does not depart from that of bacteriomes, we will only write about bacteriomes for simplicity. Basically, bacteriomes are large, elongated, paired, or unpaired structures that are localized in the insect’s body cavity in the abdomen. In males, bacteriomes are much smaller than in females and are always localized in the rearmost portion of the abdomen (not shown). In contrast, in females, bacteriomes are distributed close to the ovaries and show different spatial arrangements. Usually, they run longitudinally or transversely through the posterior part of the abdomen, but sometimes they are intertwined with the ovaries (not shown). Based on the comparative analysis of bacteriome organization, we distinguished 7 types of bacteriomes in planthoppers, differing in structure and number of symbionts inhabiting them (Figure 4). The first four types (A–D) refer to bacteriomes containing only one symbiont type (monosymbiotic). Type A bacteriome comprises several mononucleated bacteriocytes and is surrounded by a single layer of epithelial cells. The thickness of epithelium varies among bacteriomes harboring different symbionts. Type B represents plasmodium-like, nuclei-rich syncytial bacteriomes covered by the epithelium of varying thickness (similar to type A). Bacteriome types C and D are not surrounded by epithelial bacteriome sheath. Type C is made up of several bacteriocytes, whereas type D is a multi-nuclei syncytium. The remaining three types (E–G) refer to bacteriomes harboring two different microorganisms, generally with more complex organization (disymbiotic). Bacteriome type E comprises several closely adjacent bacteriocytes filled with symbiont 1, which are covered by a single syncytial bacteriome harboring symbiont 2. In turn, in bacteriome type F, two kinds of symbionts are mixed in the cytoplasms of one bacteriocyte. The last type of bacteriome (G) shows a unique structure that can be described as “cells enclosed in a cell.” The bacteriome is a big multi-nuclei cell filled with cells of symbiont 1 and bacteriocytes containing symbiont 2.

### 3.3 Ancestral symbionts *Sulcia* and *Vidania* retain conserved morphology and localization

**FIGURE 5:**
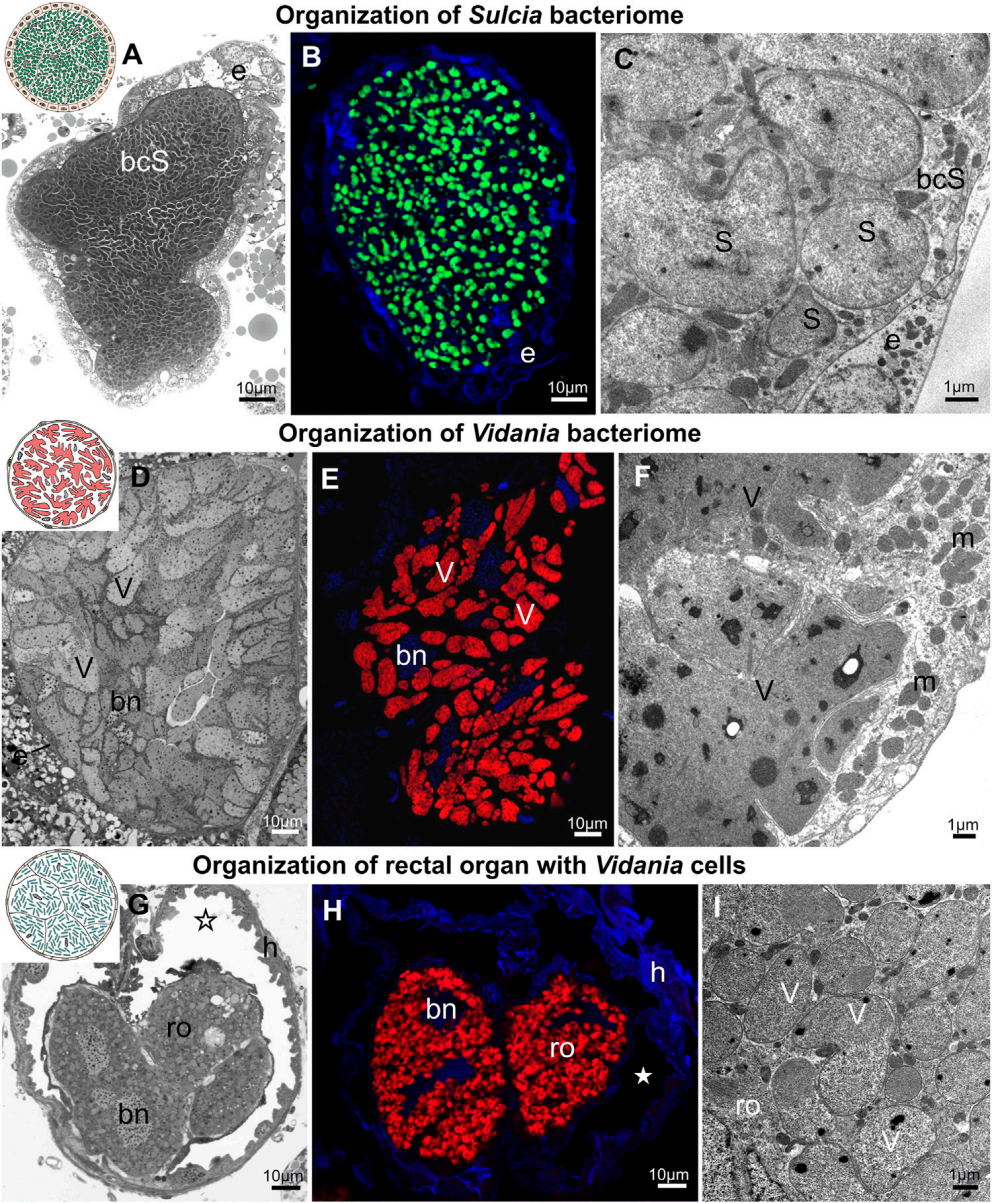
Bacteriome structure and morphology of ancient planthopper symbionts *Sulcia* and *Vidania*. **(A)** Organization of *Sulcia* bacteriome **(B)** Visualization of bacteriomes inhabited by *Sulcia* using *Sulcia*-specific probe (green), blue represents DAPI, **(C)** Ultrastructure of *Sulcia* cells. **(D)** Organization of the *Vidania* bacteriome. **(E)** Visualization of bacteriome inhabited by *Vidania* using *Vidania*-specific probe (red), blue represents DAPI **(F)** Ultrastructure of *Vidania* bacteriome **(G)** Rectal organ with *Vidania* symbiont in the hindgut lumen. **(H)** FISH detection of *Vidania* in the rectal organ. **(I)** Ultrastructure of *Vidania* cells in the rectal organ. For a detailed description of these images, see the main text. **(A,D,G)** Light microscope (LM). **(B,E,H)** Confocal microscope. **(C,F,I)** Transmission electron microscope (TEM). bcS - *Sulcia* bacteriocyte, bn - bacteriocyte nucleus, e - epithelium, h - hindgut, m - mitochondria, ro - rectal organ, asterisk - gut lumen, S - *Sulcia*, V - *Vidania*. Insect species: **(A)** *Tettigometra sulphurea* (Tettigometridae) **(B)** *Akotropis quercicola* (Achilidae) **(C)** *Meenoplus albosignatus* (Meenoplidae) **(D,E)** *Tettigometra sulphurea* (Tettigometridae) **(F)** *Ommatidiotus longiceps* (Caliscelidae) **(G)** *Proutista* sp. (Derbidae) **(H)** *Akotropis quercicola* (Achilidae) **(I)** *Cixidia pilatoi* (Cixiidae).

The two ancestral symbionts of planthoppers, *Sulcia* and *Vidania,* are restricted to bacteriomes in all examined planthopper species. These two symbionts always reside in separate bacteriomes (Figure 5). Bacteriomes occupied by *Sulcia* represent type A. They are paired, tubular, and covered by a thick monolayered epithelium termed bacteriome sheath (Figures 5A–C). The epithelial cells are cube-shaped and have large, spherical nuclei and numerous mitochondria in the cytoplasm (Figures 5A, C). The bacteriocytes that make up the bacteriome are uninucleated and closely adhere to each other. Their cytoplasm is filled with variably shaped (pleomorphic) cells of *Sulcia* (Figures 5A–C). Basically, the *Sulcia* cell shape and size are similar among planthoppers, with one exception for *Kelisia* and *Stenocranus* genera in which *Sulcia* cells are smaller and more spherical (not shown).

The *Vidania* symbiont is also strictly limited to bacteriomes (Figures 5D–I). In both sexes, bacteriomes with *Vidania* occur in the body cavity between internal organs. These bacteriomes represent type B, and are large, multi-nucleated syncytial organs surrounded by a very thin, flattened bacteriome sheath (Figures 5D, F). Their cytoplasm is tightly packed with giant, lobed *Vidania* cells (Figures 5D–F). The nuclei are usually scattered between *Vidania* cells, whereas the numerous mitochondria mostly lay in the peripheral part of the bacteriome under the bacteriome membrane (Figure 5F). In addition to these syncytial bacteriomes present in both sexes, females have an additional, unpaired bacteriome called a rectal organ, which is situated in the deep invagination of the hindgut that protrudes into its lumen. The rectal organ is composed of several binucleated bacteriocytes. The *Vidania* cells that fill them are pleomorphic, in sharp contrast to the cells in the primary bacteriome (Figures 5G–I).

### 3.4 Gammaproteobacterial symbionts usually reside in bacteriocytes

**FIGURE 6:**
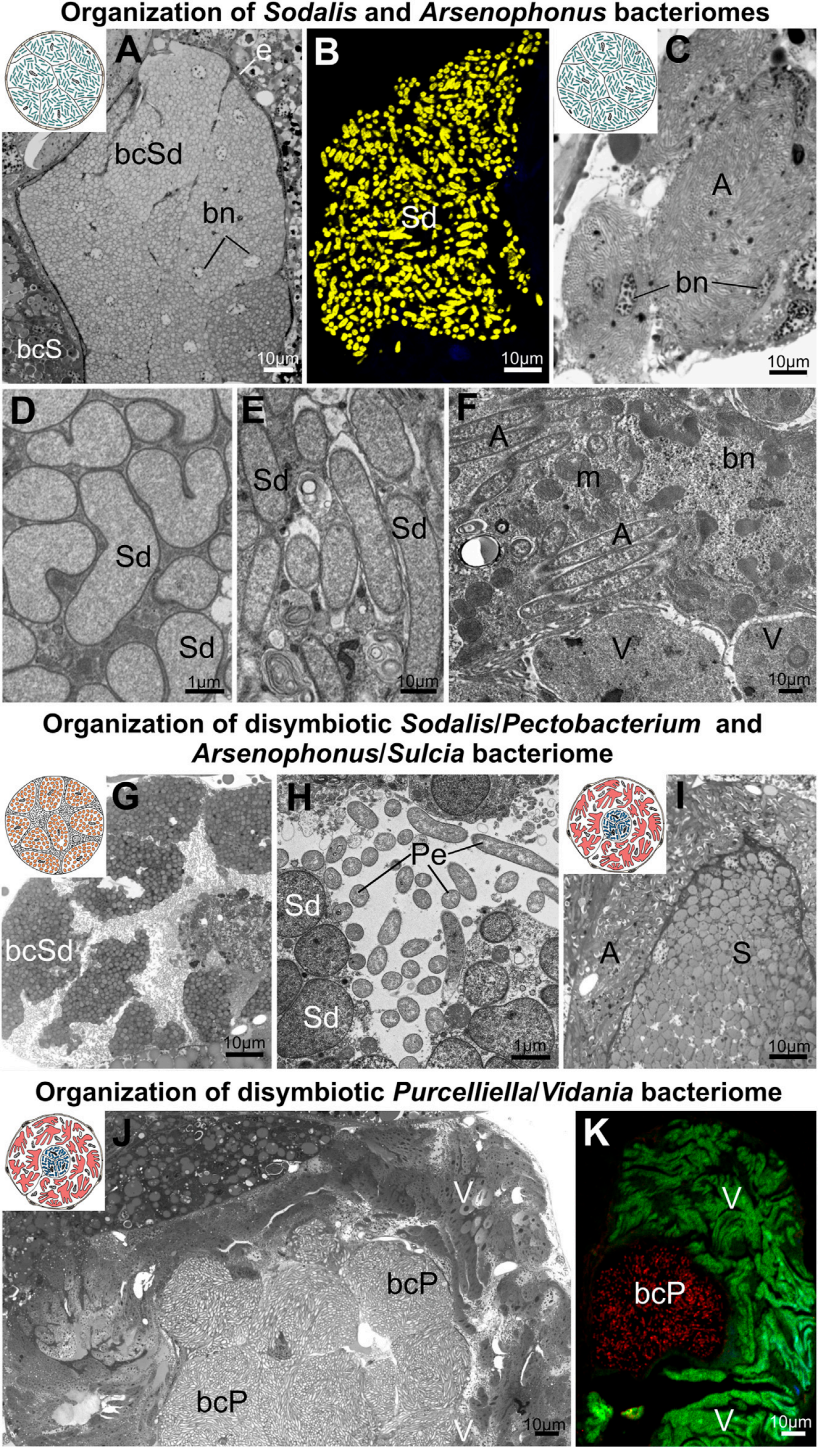
Organization of bacteriomes harboring gammaproteobacterial symbionts in planthoppers. **(A,B)** Organization of *Sodalis* bacteriome **(C)** Cluster of *Arsenophonus* bacteriocytes, **(D,E)** Ultrastructure of *Sodalis* cells **(F)** *Arsenophonus* cells in the cytoplasm of *Vidania* bacteriocyte. **(G,H)** Organization of disymbiotic *Sodalis*/*Pectobacterium* bacteriome. **(I)** Organization of disymbiotic *Arsenophonus*/*Sulcia* bacteriome. **(J,K)** Organization of disymbiotic *Purcelliella*/*Vidania* bacteriome. For a detailed description of these images, see the main text. **(A,C,G,H,I,K)** Light microscope (LM). **(B,H)** Confocal microscope. **(D,E,F,J)** Transmission electron microscope (TEM).A - *Arsenophonus*, bcP - *Purcelliella* bacteriocyte, bcS - *Sulcia* bacteriocyte, bcSd - *Sodalis* bacteriocyte, bn - bacteriocyte nucleus, m - mitochondria, Pe - *Pectobacterium*, S - *Sulcia*, Sd - *Sodalis*, V - *Vidania.* Insect species: **(A)** *Tshurtshurnella decempunctata* (Issidae) **(B)** *Tettigometra griseola* (Tettigometridae) **(C)** *Scorupella discolor* (Issidae) **(D)** *Zopherisca tendinosa* (Issiade), **(E)** *Kelisia ribauti* (Delphacidae) **(F)** *Scorupella discolor* (Issidae) **(G,H)** *Tettigometra sulphurea* (Tettigometridae) **(I)** *Pyrops clavatus* (Fulgoridae) **(J,K)** *Hyalesthes luteipes* (Cixiidae).

Gammaproteobacterial symbionts, including *Sodalis*, *Arsenophonus*, *Purcelliella,* and *Pectobacterium,* usually inhabit bacteriomes. However, the organization of these organs varies among symbiont genera and, to some extent, also host clades (Figure 6).

The most common planthopper gammaproteobacterial symbionts, *Sodalis* and *Arsenophonus,* in almost all cases, occur in separate bacteriomes (Figures 6A–C). These bacteriomes are unpaired, composed of closely adhering bacteriocytes (types C and D), and are or are not covered by an epithelial sheath. The bacteriocytes are usually binucleated and tightly packed with bacterial cells (Figures 6A, B, E, D). The exceptions from this general rule were observed in *Scorupella discolor* (Issidae), and *Pyrops clavatus* (Fulgoridae). In *S. discolor*, bacteriocytes harboring *Arsenophonus* symbionts are not integrated into compact bacteriome but form a more or less loose cluster of cells (Figure 6C). In this species *Arsenophonus* symbionts may also reside in the cytoplasm of *Vidania’s* bacteriocytes, with both symbionts mixed in the cytoplasm of the shared bacteriocytes (type G) (Figure 6F). In turn, in *P. clavatus*, bacteriocytes with *Arsenophonus* symbionts surround the *Sulcia* bacteriome (type E) (Figure 6I).

Apart from the differences in bacteriome organization, we also observed differences in *Sodalis* cell shape - from rod-shaped (in Dictyopharidae) through irregular (in Issidae) to almost spherical (in Tettigometridae) (Figures 6A, B, D, E, G, H). Furthermore, in the cytoplasm of bacteriocytes with *Sodalis* in different planthoppers species, we observed numerous lamellar bodies, which we interpret as symptoms of *Sodalis* degeneration (Figure 6E).

Other gammaproteobacterial symbionts - *Purcelliella* and *Pectobacterium*, share the bacteriome with another symbiont. *Purcelliella* - a symbiont exclusive to the family Cixiidae, occurs in a common bacteriome with *Vidania* symbiont (type E). *Purcelliella* inhabits separate bacteriocytes, but they are always covered by the large syncytial bacteriome with *Vidania* cells (Figures 6J, K). In turn, in *Tettigometra sulphurea, Pectobacterium* and *Sodalis* co-reside in the common bacteriome with the most complicated organization we observed in planthoppers (type F). This bacteriome is a large multinucleate cell; within its cytoplasm we observed *Pectobacterium* cells, as well as bacteriocytes with *Sodalis* (Figures 6G, H).

### 3.5 Alphaproteobacterial symbionts may occupy diverse tissues and organs

**FIGURE 7:**
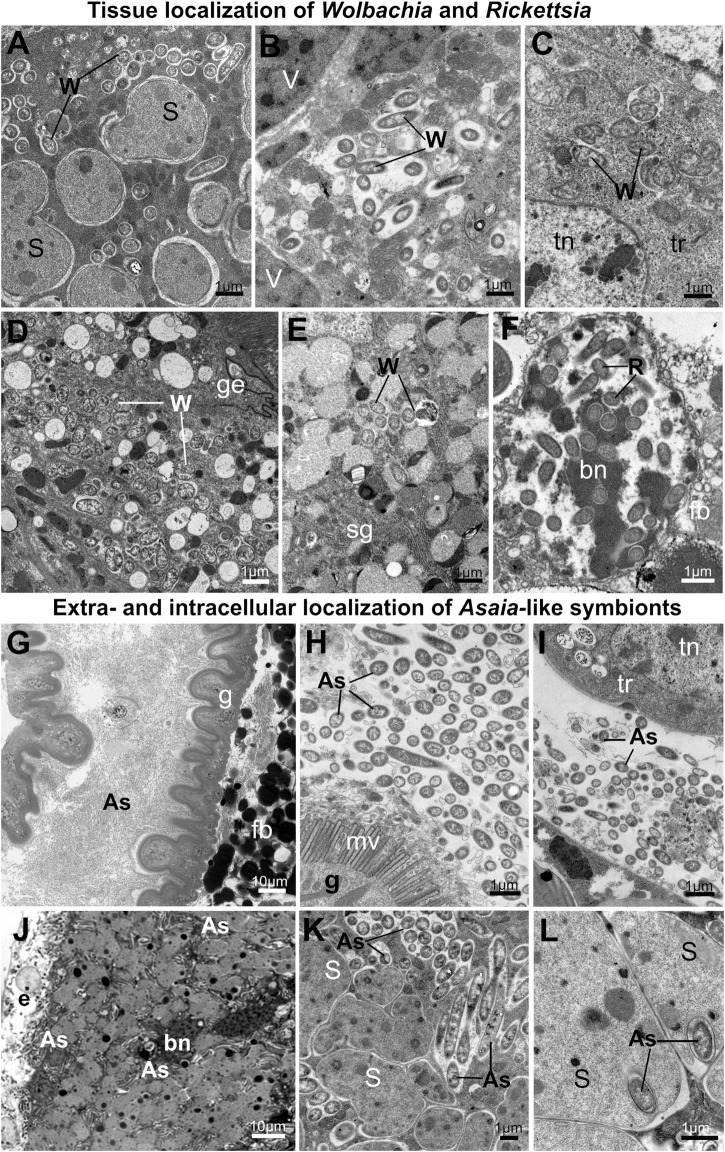
Tissue localization of alphaproteobacterial symbionts in planthoppers. (**A–F)** Tissue localization of *Wolbachia* and *Rickettsia* symbionts. **(A)** *Wolbachia* mixed with *Sulcia* cells in the shared bacteriome. **(B)** *Wolbachia* in the cytoplasm of *Vidania* bacteriocyte. **(C)** *Wolbachia* cells in the tropharium (part of the ovary). **(D)** *Wolbachia* in the gut epithelium **(E)** *Wolbachia* in the salivary gland **(F)** *Rickettsia* in the nucleus of fat body cell. (**G–L)** Extra- and intracellular localization of *Asaia*-like symbionts. **(G,H)** *Asaia*-like symbionts in the gut lumen. **(I)** *Asaia*-like cells in the hemolymph. **(J)** Bacteriome harboring *Sulcia* and *Asaia*-like symbionts **(K)** *Sulcia* cells and *Asaia*-like symbionts in the cytoplasm of shared bacteriocyte. **(L)** *Asaia*-like cells surrounded by *Sulcia* projections. For a detailed description of these images, see the main text. **(A–F,H,I,K,L)** Transmission electron microscope (TEM). **(G,J)** Light microscope (LM). As - *Asaia*-like symbiont, bn - bacteriocyte nucleus, e - epithelium, fb - fat body cell, fn - fat body cell nucleus, g- gut, ge - gut epithelium, mv - gut microvilli, R - *Rickettsia*, S - *Sulcia*, sg—salivary gland, tn - trophocyte nucleus, tr - tropharium, W - *Wolbachia.* Insect species: **(A)** *Dictyophara pannonica* (Dictyopharidae) **(B)** *Hyalesthes luteipes* (Cixiidae) **(D)** *Pentastira rorida* (Cixiidae) **(E)** *Ommatidiotus longiceps* (Calliscellidae) **(F)** *Orosanga japonica* (Ricaniidae) **(G,I)** *Phantia subquadrata* (Flatidae) **(J–L)** *Trypetimorpha occidentalis* (Tropiduchidae).

Alphaproteobacterial symbionts of planthoppers include the bacteria *Rickettsia*, *Wolbachia*, *Sphingomonas,* and bacteria related to *Asaia*. Their localization is not restricted to the bacteriomes - they may occur in other insects’ organs and tissue. Besides bacteriomes, we found alphaproteobacterial symbionts also in the cytoplasm of fat body cells, in the nuclei, gut epithelium, salivary glands, and in females, in different parts of the reproductive system, which is probably related to the symbionts’ transovarial transmission between generations. Alphaproteobacterial symbionts are also the only ones occurring extracellularly in the gut lumen and hemolymph (Figure 7).

The most common alphaproteobacterial symbionts in planthoppers - *Wolbachia* and *Rickettsia,* usually co-occur in the bacteriocytes with *Sulcia* and *Vidania*. The organization of these bacteriomes is similar to the organization of bacteriomes inhabited by *Sulcia* and *Vidania* (type A and B), respectively. The only difference is that in the bacteriocyte cytoplasm, two types of symbionts are mixed (Figures 7A, B, J, K). Alphaproteobacterial symbionts may also be dispersed in the fat body tissue. Symbionts usually occupy the cytoplasm of fat body cells, but their abundance and density differ between species. *Wolbachia* and *Rickettsia* localized in the cytoplasm of fat body cells are not very numerous and do not occur in all cells. *Rickettsia* associated with the planthopper *Orosanga japonica* (Ricaniidae) has a unique localization: we found it exclusively in the nuclei of fat body cells. In all specimens of that species examined, we observed several *Rickettsia* cells inside the nuclei (Figure 7F).

Among alphaproteobacterial symbionts detected in planthoppers, *Asaia*-like symbionts show the greatest diversity of tissue localizations across host insect species. In *P. subsquadrata* and *Z. tendinosa*, they inhabit mainly the gut lumen but are also found in hemolymph (Figures 7G–I). In turn, in *T. occidentalis*, *Asaia*-like symbionts occur exclusively in the bacteriocytes with *Sulcia* (Figures 7J–L). Most of its cells are localized in the bacteriocytes’ cytoplasm among *Sulcia* cells (Figures 7J, K). However, some are almost completely surrounded by *Sulcia* cell projections, making the localization similar to nested symbiosis observed in other Auchenorrhyncha species (Figure 7L).

### 3.6 Fungal symbionts occur in the mycetomes or fat body cells

**FIGURE 8:**
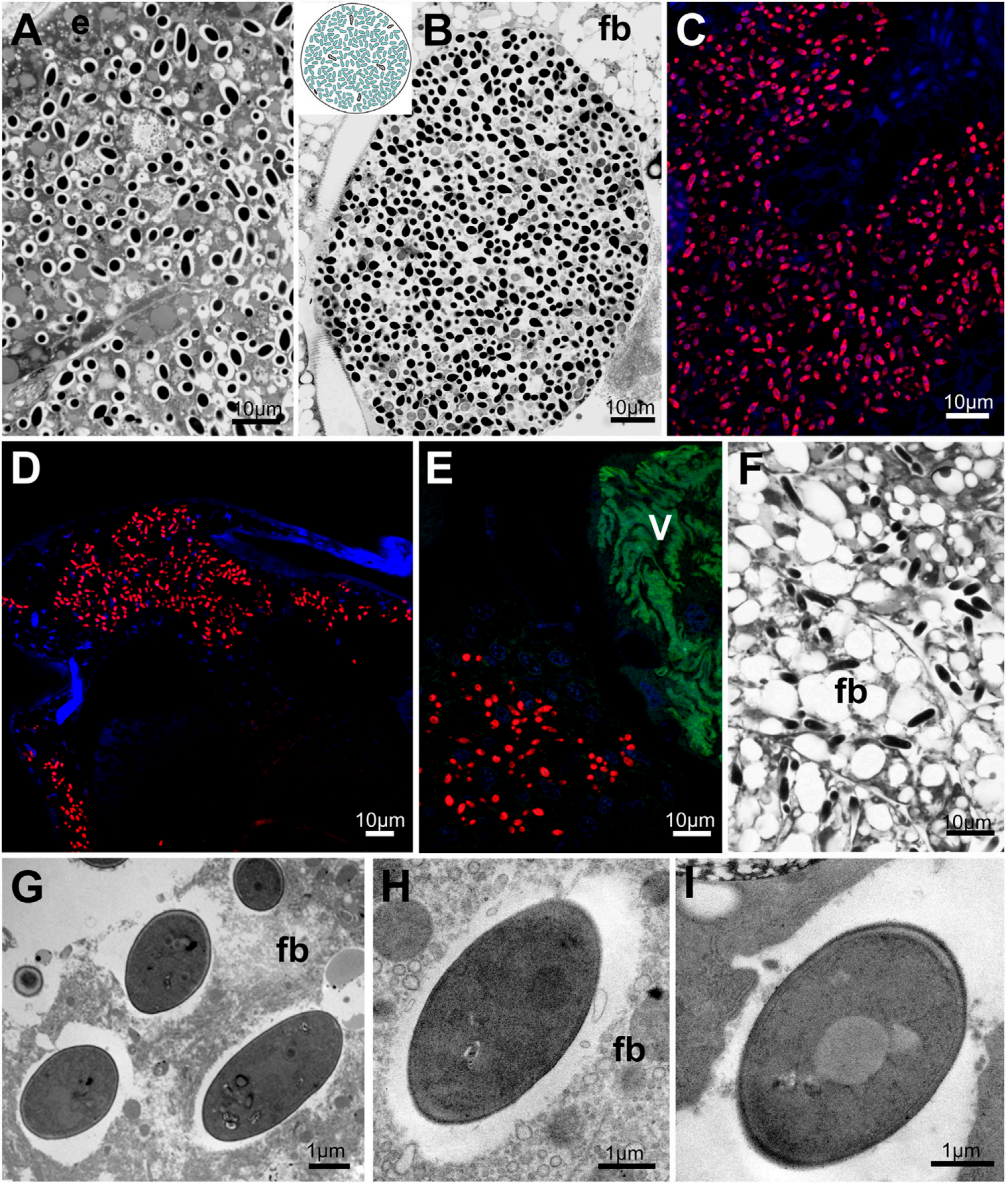
Distribution of fungal symbionts within planthopper tissue. **(A,B)** Mycetocyte with fungal symbionts. **(C)** Group of fat body cells occupied by fungal symbionts. **(D–F)** Fungal symbionts dispersed within fat body cells. **(G–I)** Ultrastructure of fungal symbiont cells. For a detailed description of these images, see the main text. **(A,B,F)** Light microscope (LM). **(C,D,E)** Confocal microscope. **(G–I)** Transmission electron microscope (TEM). **(E)** epithelium, fb - fat body, V - *Vidania.* Insect species: **(A)**. *Metcalfa pruinosa* (Flatidae) **(B)** *Phantia subquadrata* (Flatidae) **(C)** *Chloriona glauscesens* (Delphacidae) **(D)** *Conomelus* sp. (Delphacidae) **(E,F)** *Issus coleoptratus* (Issidae) **(G)** *Chloriona glauscesens* (Delphacidae) **(H)** *Dicanotropis hamata* (Delphacidae) **(I)** *Ricanula unica* (Ricaniidae).

All fungal symbionts detected in examined planthoppers species belong to the order *Hypocreales* (Figure 1). However, they colonize different tissues across the surveyed insect host species (Figure 8). Fungal symbionts associated with Flatidae inhabit large organs termed mycetomes. They are usually large, multinuclear syncytia (type B and D) filled with fungal cells and sometimes surrounded by a one-layered epithelium (Figures 8A, B, G). In contrast, fungal symbionts present in members of families Ricaniidae, some Delphacidae, and *I. coleoptratus* from the family Issidae are not segregated to the mycetomes but occupy fat body cells. They may be scattered across the whole fat tissue (like in all Ricaniidae, *N. distincta* and *I. coleoptratus*) (Figures 8D, E, F, H) or occupy clusters of fat body cells located between the internal organs and against the body wall (Figure 8C). In *O. japonica* (Ricaniidae), fungal symbionts also show intercellular localization in hemolymph between fat body cells (Figures 8F, I).

All fungal symbionts have similar morphology - their cells are ellipsoidal and surrounded by a thick cell wall (Figures 8G–I).

## 4 Discussion

Heritable endosymbiotic systems of Auchenorrhyncha are much more diverse than most other insects, and their comprehensive analysis requires using many different tools, including sequencing-based techniques and modern microscopy. Here, by combining metagenomics and microscopy, we showed that out of 44 species representing 15 planthopper families, 34 hosted ancient bacterial nutritional endosymbionts *Vidania* and (usually) *Sulcia*, which have been replaced by *Hypocreales* fungi in the remaining species. These symbionts that likely play key nutritional roles are usually accompanied by additional bacteria - *Gammaproteobacteria*, *Alphaproteobacteria*, or sometimes *Actinobacteria*, and *Tenericutes*. For these microbes, we have identified and described seven tissue localization patterns; furthermore, for bacteriocytes/mycetocytes where microbes reside most often, we identified seven different types of organization. In the sections below, we discuss symbiont distribution and replacement patterns in relation to what is known about their functions; then, we consider the diversity of symbiont tissue localizations and bacteriome/mycetome structures.

### 4.1 A snapshot of the diversity of anatomical integration patterns of insect symbionts

Microorganisms associated with the taxonomic diversity of insects show a wide range of tissue localizations and morphologies, depending on the nature of their associations with hosts. The range of tissue localizations of endosymbiotic microorganisms we report from planthoppers overlaps substantially with patterns known from other insect-microbe endosymbioses, despite the taxonomic diversity of partners and independent origins of these associations.

The localization of the majority of planthopper endosymbionts with putative nutritional roles is restricted to bacteriocytes - insect cells adapted to gathering and storing the symbionts. Basically, these symbionts share two common features: they play a nutritional role in host biology and are vertically transmitted between insect generations. The bacteriocyte-associated symbionts have been reported so far in six insect orders, including Hemiptera, Blattodea, Coleoptera, Diptera, Hymenoptera, and Psocodea (Buchner, 1965; Douglas, 2022). The independent origins of these symbioses (McCutcheon et al., 2019) and dramatic biological differences among and within these insect orders translate to a massive variety of body localizations and organizations of bacteriomes.

We showed that in planthoppers, like in beetles, cockroaches, and other hemipterans, bacteriocytes/bacteriomes are usually distributed in the body cavity within fat body tissue, usually close to reproductive organs or intestine (Buchner, 1965; Michalik et al., 2014a; Michalik et al., 2014b; Michalik et al., 2018a; Michalik et al., 2018b; Hirota et al., 2020; Latorre et al., 2022). In contrast, in *Camponotus* ants, Hippoboscoidea flies, and some sucking lice, bacteriocytes are directly associated with the intestinal wall. They might be inserted between gut epithelium and muscle layer (in Hippoboscoidea flies and sucking lice) or between gut epithelial cells (in *Camponotus* ants) (Buchner, 1965; Schröder et al., 1996; Nishide et al., 2022). In turn, in tsetse flies, paired bacteriomes are localized in the midgut lumen (Rio et al., 2012), resembling the localization of the rectal organ in females of *Vidania*-hosting planthopper species, but to our knowledge, not reported so far from any other insect group.

The two basic bacteriome types we report from planthoppers, monosymbiotic and disymbiotic, are both known from other insects. Monosymbiotic bacteriomes, bearing a single symbiont taxon and with relatively simple organization, are common in insects (Buchner, 1965). Some of the better-known examples include cockroaches (*Blattobacterium*), tsetse flies (*Wigglesworthia*), aphids (*Buchnera*), and scale insects (*Tremblaya phenacola*, *Kotejella*) (Rio et al., 2012; Michalik et al., 2014b; Michalik et al., 2018b; Michalik et al., 2019; Latorre et al., 2022). The less common are disymbiotic bacteriomes harboring two different types of microorganisms. Usually, such intimate relationship occurs between co-primary symbionts that complement their nutritional functions. The best examples are bacteriomes containing *Sulcia* and its companion symbiont (*Nasuia*, *Zinderia,* or *Hodgkinia*) that occupy distinct regions of a single bacteriome in planthopper’s sister group, Cicadomorpha (Koga et al., 2013; Kobiałka et al., 2018; Łukasik et al., 2018). However, disymbiotic bacteriomes were observed in Hemiptera other than Auchenorrhyncha, including some aphids, psyllids, whiteflies, scale insects, and bed bugs (Hosokawa et al., 2010; Nakabachi et al., 2013; Michalik et al., 2014b). In all these groups, ancestral symbionts share the common bacteriome or even bacteriocyte with more recently acquired microorganisms. For example, in the psyllid *Diaphorina citri*, *Carsonella* and *Profftella* are localized in different cells that form the common bacteriome, with a very similar organization to the bacteriome harboring *Pectobacterium* and *Sodalis* in the planthopper *T. sulphurea* (Nakabachi et al., 2013). In other cases, two types of microorganisms co-reside in the cytoplasm of a single bacteriocyte, like in the bed bug *Cimex pectoralis* when *Wolbachia* share the bacteriome with gammaproteobacterial symbiont (Hosokawa et al., 2010), in weevil *Sitophilus oryzae* when it coexists in common cells with obligatory symbiont (Heddi et al., 1999) or in the whitefly *Bemisia tabaci* possessing usually two (*Portiera* and *Hamiltonella*), but in some individuals, up to six endosymbionts in the cytoplasm of a single bacteriocyte (Gottlieb et al., 2008). The most unusual symbiont distribution we demonstrated in planthoppers is the intracellular localization of *Asaia*-like symbiont within *Sulcia* cells. Such spatial organization of dual symbiosis was noted previously in mealybugs from the Phenacoccinae family and two leafhopper species in which gammaproteobacteria reside in *Tremblaya* or *Sulcia* cells, respectively (Michalik et al., 2014b; Kobiałka et al., 2018; Garber et al., 2021).

In planthoppers as well as other insects, endosymbiont tissue localizations outside of bacteriomes - in fat body cells or extracellularly - are more common for facultative and recently established symbioses. These microbes vary in their tissue tropism in contrast to long-term associates. The colonization of the cytoplasm of fat body cells has been demonstrated several times for widely distributed facultative endosymbionts *Wolbachia* and *Rickettsia* (Gottlieb et al., 2008; Pietri et al., 2016; Kobiałka et al., 2018) or *Ophiocordyceps* symbionts in some scale insects, aphids and auchenorrhynchans (Fukatsu and Ishikawa, 1992; Matsuura et al., 2018; Michalik et al., 2021). Uniquely, facultative symbionts can colonize the cell nuclei, as reported for *Rickettsia* and *Wolbachia* in a few auchenorrhynchans (Arneodo et al., 2008; Kobiałka et al., 2018, this paper).

The comprehensive understanding of the relationship between tissue localization and host and symbiont phylogenetic placement and function would require more systematic studies combining microscopy and sequencing of the type conducted in a few insect clades to date (Salem and Kaltenpoth, 2022). Nevertheless, the general patterns we report from planthoppers seem to be fairly universal across the variety of insects.

### 4.2 Symbiont acquisition and replacement as the main driving forces of the planthopper symbionts’ diversity

Essential endosymbiotic associations can be very stable, as evidenced by the long-term conservation of the organization and function of cellular organelles. However, recent research has made it clear that in many organisms, endosymbiosis is an ongoing and dynamic process that strongly influences their biology (McFadden, 2001; Poole and Gribaldo, 2014; Husnik and McCutcheon, 2016; Matsuura et al., 2018; Michalik et al., 2021). Planthoppers serve as a good example of such dynamic processes and patterns. Like almost all other Auchenorrhyncha, planthoppers rely on the supplementation of their nutritionally imbalanced diet on symbioses dating back some 300 my (Moran et al., 2005; Bennett and Mao, 2018; Michalik et al., 2021). However, the current picture of symbiosis in many Fulgoromorpha departs significantly from the ancestral state.

We showed that the ancient symbionts *Sulcia* and *Vidania* are still present in most planthopper families and a large share of species, detecting *Vidania* in 77% of species examined and *Sulcia* in 66%. These values are higher than Müller (1949) microscopy-based estimates of 58% and 41%, respectively, and Urban and Cryan’s (2012) diagnostic PCR-based estimates (52% and 39%). The discrepancies likely result from different sampling depths across families, combined with sampling different geographic regions, and perhaps methodological biases. Regardless, it is clear that the loss of one or both of the ancestral symbionts took place many times in the evolutionary history of planthoppers and was usually coupled with the acquisition of new microorganisms that took over their biological functions (Urban and Cryan, 2012; Fan et al., 2015).

The replacement of *Sulcia* by *Ophiocordyceps* fungi while *Vidania* is retained, observed in *I. coleoptratus* (Issidae), parallels the observations from some Deltocephalinae and Ledrinae leafhoppers and from cicadas (Nishino et al., 2016; Kobiałka et al., 2018; Matsuura et al., 2018). In cicadas, specialized fungal pathogens replaced the *Hodgkinia* symbiont independently in different clades, taking over its nutritional responsibilities, while *Sulcia* remained in place (Matsuura et al., 2018). However, in planthoppers, fungal replacement of both *Vidania* and *Sulcia* seem to be more common than the replacements of *Sulcia* alone. We found Hypocreales fungi, without *Sulcia* and *Vidania* but typically accompanied by other bacteria, in five planthopper families: Acanalonidae, Flatidae, Ricaniidae, and Delphacidae (in subfamily Delphacinae only). Symbiotic systems with fungal symbionts playing the central role were previously documented in Hemiptera other than planthoppers, including the leafhopper *Scaphoideus titanus*, some aphids (Hormaphididae), and scale insects (Coccidae) (Noda et al., 1995; Michalik et al., 2009; Fan et al., 2015; Szklarzewicz et al., 2021).

The loss of *Sulcia* that we observed in families Derbidae and Achilidae, not compensated by the acquisition of other nutrient-providing symbionts, is harder to explain. It seems possible that this monosymbiotic system, unique among Auchenorrhycha (Müller, 1949), is related to the special habitat and food preferences of these planthoppers, thought to feed on fungal hyphae during their larval stage (Howard et al., 2001). Such a diet may be more nutritionally balanced than plant sap, eliminating the need to have all essential amino acids provided by the symbionts, and enabling the loss of symbionts that have thus become redundant. The loss of *Sulcia* may be less detrimental for planthoppers than losing *Vidania*, which provides 7 out of 10 essential amino acids (Bennett and Mao, 2018; Michalik et al., 2021; Deng et al., 2022). It is also possible that *Wolbachia*, present in all Derbidae planthoppers that we characterized, contributes to nutrition, as it does in bed bugs and perhaps, based on genomic data, in some Dictyopharidae planthoppers (Hosokawa et al., 2010; Michalik et al., 2021). However, the diffuse localization of *Wolbachia* in the insect body and its low abundance in bacteriocytes, the host cells that mediate metabolic exchanges, do not support the hypothesis of a significant nutritional role for the symbiont.

In fact, microbes other than *Vidania*, *Sulcia,* and/or fungi, are often likely to contribute to planthopper nutrition. Gammaproteobacterial symbionts are the strongest candidates for important nutritional roles. In all four planthopper symbioses characterized to date using genomics approaches, the genomes of gammaproteobacterial symbionts (*Purcelliella*, *Sodalis*, *Arsenophonus*) encoded B vitamin biosynthesis genes (Bennett and Mao, 2018; Michalik et al., 2021) and some amino acid biosynthesis genes. This was despite their independent origins and distinct genomic characteristics, indicative of very different histories of association with hosts. This seems to be a more general trend: some clades of *Gammaproteobacteria* have repeatedly colonized diverse planthoppers, adopting means of transmitting vertically, providing deficient nutrients, and replacing symbiont strains that were there before. The genus *Sodalis* is a particularly striking example. It comprises both versatile opportunists capable of infecting humans but encoding an array of biosynthesis genes, and heritable endosymbionts of diverse insects that are likely derived from such opportunists (Enomoto et al., 2017; McCutcheon et al., 2019). For instance, Husnik and McCutcheon (2016) proposed that the diversity of genomic characteristics of mealybug endobacterial symbionts inhabiting the cytoplasm of their ancient *Tremblaya* endosymbionts, often related to and likely derived from *Sodalis*, indicates their independent origin and convergence on supplementing insect hosts with deficient nutrients. These patterns seem to be repeated among diverse gammaproteobacteria infecting 57% of the surveyed planthoppers. Some of them seem to form relatively long-term associations with hosts, exemplified by *Purcelliella* in the family Cixiidae. Other associations may be more recent or even largely transient, as evidenced by symbiont genus-level distribution on the host phylogeny, and especially intra-species infection polymorphism revealed using 16S rRNA amplicon sequencing (Figure 2). In many cases, however, it is impossible to conclude about the stability of the association based on the currently available data. McCutcheon et al. (2019) presented the challenges associated with phylogenetic reconstructions in these symbionts, whose genomes seem to undergo the spiral of rapid degeneration following the independent establishment in different hosts, with corresponding massive variations in evolutionary rates. Confident reconstruction of the evolutionary histories of these symbioses would require much more systematic sampling and genome-level datasets.

Associations of planthoppers with alphaproteobacterial symbionts seem to be less stable than symbiosis with *Gammaproteobacteria*. Observed in 35% of the surveyed planthopper species, these bacteria are not always fixed within populations. Facultative endosymbionts, the functional category that many strains of *Wolbachia* and *Rickettsia* are assigned to, are characterized by their patchy distribution across and within insect clades and species, variable prevalence within populations, and ability to occasionally transmit horizontally in addition to vertical transmission (Heath et al., 1999; Ahmed et al., 2016). However, in at least some cases, they have formed stable or even obligatory associations with hosts (Hosokawa et al., 2010). On the other hand, *Acetobacteraceae* live in a range of environments (Komagata et al., 2014), and the association of genus *Asaia* with flowers as well as guts of mosquitoes and other insects suggests its frequent environmental transmission (Favia et al., 2007; Bassene et al., 2020). This may not always be the case, given its highly specific endobacterial localization in *T. occidentalis*. This unique association of *Sulcia* with *Asaia*-like symbiont requires deeper analyses and will be the subject of a subsequent study.

Combined, the existing data make it clear that the impressive diversity of symbioses across planthoppers is a combination of their long-term co-diversification with hosts and frequent independent infections with a few clades of bacteria and fungi. Following such infections, likely often leading to the replacement of previously colonizing microbes, the stability of the association and its subsequent evolution may vary. Our ongoing genomics work on microbes associated with these and other planthoppers could reveal the nature and biological significance of many of the symbiotic associations.

### 4.3 Conserved nature of established symbioses

In four auchenorrhynchan superfamilies, beta- or alphaproteobacterial symbionts have established different tissue localizations relative to their co-symbiont *Sulcia*, also dividing nutritional responsibilities differently (McCutcheon and Moran, 2010; Mao et al., 2018; Łukasik et al., 2018; Michalik et al., 2021). In all Cicadomorpha, *Sulcia* and its co-symbiont generally occupy different regions of the same bacteriome; *Sulcia* colonizes the outer portion of the bacteriome, and its partner is localized in the bacteriome’s central part. In planthoppers, our microscopic survey across families separated by up to about 200 my of evolution revealed that *Sulcia* and *Vidania* always occupy separate bacteriomes. However, within a superfamily, as long as both ancestral symbionts are present, their nutritional functions and the organization of symbiont–containing tissue appear highly conserved (Müller, 1940a; Müller, 1940b; Buchner, 1965; Koga et al., 2013; Kobiałka et al., 2018; Michalik et al., 2018a; Łukasik et al., 2018; Michalik et al., 2021). As explained earlier, planthopper bacteriome size, shape, and localization within the abdominal cavity vary across species and between males and females of the same species. However, generally, there is no difference in the internal organization of bacteriome among sexes, as also reported from other auchenorrhynchans (Kobiałka et al., 2018; Szklarzewicz et al., 2020). The situation is different with the rectal organ, the second type of *Vidania* bacteriome found exclusively in females, which contain morphologically different *Vidania* cells. Given *Vidania* shape similarity to that observed during transovarial transmission, it has been proposed that this pool of *Vidania* cells is intended for the transmission to the progeny (Buchner, 1965; Bressan and Mulligan, 2013).

The only observed departures from the universal - ancestral organization were when other, more recently acquired symbionts established residence within *Sulcia* or *Vidania* bacteriomes. For example, in the family Cixiidae, *Vidania* always shares a common bacteriome with *Purcelliella*, whereas in *P. clavatus, Sulcia* co-occurs with *Sodalis*. A unique example of such integration of a newly acquired symbiont in the biology of ancient one is *T. occidentalis*, where *Asaia*-like symbiont established residence within *Sulcia* cells. In the three planthopper clades characterized to date using genomics, different combinations of independently acquired, accessory symbionts seem to have had limited effects on the genomes or functions of the ancient symbionts (Bennett and Mao, 2018; Michalik et al., 2021; Deng et al., 2022). However, it remains to be demonstrated whether and how *Sulcia* and *Vidania* have been affected in some of the more complicated associations, including those described here.

### 4.4 Idiosyncracy in newly forming symbioses

Across our 44 planthopper species, we observed multiple types of the organization of tissues occupied by accessory symbionts representing different clades and derived from independent infections. Some of these distribution patterns closely resemble observations from other insects. For example, gammaproteobacterial symbiont *Arsenophonus* usually occupies distinct bacteriomes. We found this type of organization in 7 out of 8 planthopper species harboring this symbiont, and it was previously reported from other hemipterans, including leafhoppers, cicadas, whiteflies, and scale insects (Gottlieb et al., 2008; Kobiałka et al., 2018; Michalik et al., 2018b; Huang et al., 2020; Huang et al., 2023). Other organization types are very unusual, and perhaps unique to planthoppers. For example, in *T. sulphurea, Pectobacteria* share the common bacteriome with another accessory symbiont - *Sodalis*, creating a bacteriome with a structure never before reported from Auchenorrhyncha or, to our knowledge, any other insect.

Closely related microbes may settle within different tissues when colonizing different hosts. For example, in most planthoppers, bacteria *Sodalis* inhabit separate bacteriomes, but sometimes they colonize the same organs as other symbionts, including *Sulcia* or *Pectobacterium*. Likewise, *Ophiocordyceps* fungi may be localized in mycetomes, or alternatively, dispersed in fat body cells. Both these widely distributed microbial clades, when colonizing other insects, have established in a wide range of tissues. Indicatively, *Sodalis*, depending on the host, may be localized in the bacteriocytes, gut lumen, gut epithelium, milk gland, or even inside the cytoplasm of other symbiotic bacteria (Attardo et al., 2008; Masson et al., 2015; Husnik and McCutcheon, 2016; Michalik et al., 2021). Similarly, *Ophiocordyceps,* which replaced *Hodgkinia* in different clades of Japanese cicadas, established either within an epithelium of the shared *Sulcia-Hodgkinia* bacteriome (resulting in a shared *Ophiocordyceps-Sulcia* bacteriome), or in a new type of bacteriome while *Sulcia* remained on its own (Matsuura et al., 2018).

The symbiont localization may also be pre-determined to at least some extent by the host internal environment, which is likely to vary consistently among host clades. A great example of such pre-determination are mealybugs - where independently established gamma-symbionts across multiple host species always localized inside the cells of *Tremblaya* (Husnik and McCutcheon, 2016). In planthoppers, we need both broader sampling and robust phylogenomic tools before talking about differences among host clades in the tissues where independently acquired symbionts localize. Then, an important question is how this diversity of tissue localizations is shaped and what determines the localization and distribution of microbes newly acquired by different host clades. It is likely that the localization is a balance between pre-adaptations in different microbial clades or strains, pre-adaptations in different insect lineages, evolutionary processes in both symbionts and hosts, and likely, a good deal of chance. Following a new colonization, the microbe - likely a pathogen failing to display virulent phenotype, or a versatile opportunist, faces multiple challenges, among the most important of which are avoiding insect’s immune system response and colonizing its tissues (Bright and Bulgheresi, 2010). The initial localization of a newly arrived symbiont may also be determined by the mode of infection, as it may need to pass through multiple tissues to colonize the host. However, following successful transmission to subsequent generations, it is likely to establish within a certain tissue. We think that in the longer term, that localization is unlikely to change spontaneously. However, over time, evolutionary processes acting on the host and symbiont genomes, including the progressing symbiont genome reduction and their increasing dependence on host genome-encoded mechanisms, as well as other changes to the host biology and ecology, could change the nature of interaction and the organization of cells and tissues where the symbiont is localized. The structural diversity of mitochondria and chloroplasts across eukaryotic clades and tissues could serve as an example (Roger et al., 2017; Choi et al., 2021).

Conversely, localization may determine symbiont biology and evolution, including long-term prospects of a newly established infection. For example, *Purcelliella*, a *Sodalis*-allied symbiont of the Cixiidae planthoppers, stands out from among related strains by its greatly reduced genome (Bennett and Mao, 2018) and presence in all surveyed members of the family - both suggestive of an unusually stable association. It is tempting to speculate that this stability is related to its unique localization in bacteriomes shared with *Vidania*.

### 4.5 The importance of a multi-pronged characterization of insect symbiosis diversity

The knowledge of the symbiont diversity in planthopper tissues is not new. However, we have gone a long way since the times of Müller (1940a), Müller (1940b) and Buchner (1965), reconstructing the co-diversification and replacement patterns in auchenorrhynchan symbionts using relatively simple microscopy techniques, yet still with impressive accuracy. The rapid development of sequencing-based techniques has greatly simplified the task of characterizing host-symbiont associations. Through a combination of broad sampling and cost-effective screens, we can uncover the general patterns of symbiont diversity, distribution, and stability within clades. Shotgun metagenomics enables high-resolution phylogenomic reconstructions of host-symbiont relationships and informs us of putative symbiont functions. Transcriptomics and proteomics are powerful approaches to verify these functions. Finally, all these approaches can be combined with genetic manipulations and experiments to unequivocally demonstrate the nature and significance of broadly relevant processes (Bublitz et al., 2019; Su et al., 2022).

Unfortunately, such combinations of cutting-edge tools have not been applied to many systems, and outside of a few model organisms, knowledge about the diversity, distribution, evolutionary patterns, and biological significance of symbiotic microorganisms is lacking. Symbiont distribution within host tissues is also among these understudied areas, despite being critical for understanding the nature of host-symbiont interactions. The current study shows how comparative microscopy can complement increasingly popular sequencing-based approaches, in addressing a series of questions about how the symbioses are organized, how they function, and how they evolve. As we proceed in our attempts to describe the diversity and nature of host-microbial associations and their stability and roles in our rapidly changing world, microscopy and sequencing will form a particularly powerful combination of research tools.

## Data Availability

Sequence data have been deposited in GenBank under accession numbers provided in Supplementary Table S4 and at https://github.com/AnnaMichalik22/Bacteriome-organization-in-planthoppers.git.

## Supplementary material

SUPPLEMENTARY TABLE S1 Collection and processing details of insect populations used in that study.

SUPPLEMENTARY TABLE S2 Protocols.

SUPPLEMENTARY TABLE S3 OTUs table based on 16S rRNA amplicon data for replicate individuals of the same species.

SUPPLEMENTARY TABLE S4 NCBI accession numbers for the sequences and datasets.
